# Radiocesium-bearing microparticles found in dry deposition fallout samples immediately after the Fukushima nuclear accident in the Kanto region, Japan

**DOI:** 10.1038/s41598-023-49158-2

**Published:** 2023-12-09

**Authors:** Yuki Takaku, Shogo Higaki, Masahiro Hirota, Hiroyuki Kagi

**Affiliations:** 1 Geochemical Research Center, Graduate School of Science, The University of Tokyo, 7-3-1 Hongo, Bunkyo-ku, Tokyo, 113-0033 Japan; 2 Isotope Science Center, The University of Tokyo, 2-11-16 Yayoi, Bunkyo-ku, Tokyo, 113-0032 Japan; 3 Research Center for Supports to Advanced Sciences, Shinshu University, 3-1-1 Asahi, Matsumoto, Nagano 390-8621 Japan

**Keywords:** Environmental sciences, Environmental impact

## Abstract

Radiocesium released by the Fukushima Dai-ichi Nuclear Power Plant (FDNPP) accident still exists in the environment in two forms: adsorbed species on mineral particles in the soil and microparticles containing radiocesium mainly composed of silicate glass (CsMPs). CsMPs are dispersed not only around the FDNPP but also over a wide area of the Kanto region. The behavior and characteristics of CsMPs must be investigated to evaluate the impact of the FDNPP accident. Deposited particles including radiocesium were wiped from metal handrails on balconies and car hoods using tissue papers at six locations in the Kanto region (Tokai village, Ushiku City, Abiko City, Chiba City, Kawaguchi City, and Arakawa Ward) between March 15 and 21, 2011. CsMPs were isolated from the samples, and their characteristics were investigated. In total, 106 CsMPs derived from Unit 2 were successfully separated from 13 tissue paper samples. The radiation images of the two types of CsMPs discovered in Ushiku City demonstrate that CsMPs can easily become susceptible to fragmentation over time, even in the absence of weathering effects.

## Introduction

The Fukushima Dai-ichi Nuclear Power Plant (FDNPP) accident released a large amount of radioactive materials into the environment. Among them, radiocesium affects the environment because of its long half-life. Radiocesium has been believed to be released into the environment by the accident in two forms. One is a soluble aerosol state, which accounts for the majority of the released radiocesium. Radiocesium released from the FDNPP was carried with sulfate aerosols in the atmosphere and then deposited on the ground. It was reported that approximately 95% of the released radiocerium was deposited on forests, vegetable fields, and rice paddies^1^. The other form is an insoluble particle that is believed to have been released directly from a nuclear reactor damaged by meltdown or hydrogen explosion; these particle, which were first detected in Tsukuba City, Ibaraki Prefecture, approximately 170 km southwest of the FDNPP, are called radiocesium-bearing microparticles (CsMPs) and have been characterized by a variety of methods^2^. CsMPs are a unique form of radiocesium formed by the FDNPP accident. The ratio of the radioactivity of CsMPs to that of soluble radiocesium has been reported^3–8^. These studies show that the amount of Cs released in a soluble state was the most significant, but a substantial amount of CsMPs was also released.

There are two types of CsMPs, Type A and Type B, which differ in shape, size, and ^134^Cs/^137^Cs radioactivity ratio^2,3,9,10^. Type A particles were emitted from Units 2 and 3 and can be spherical or non-spherical with a particle size of 0.5–10 µm. The ^134^Cs/^137^Cs radioactivity ratio of the inventory in Unit 2 is estimated to be 1.08, and that in Unit 3 is 1.05^11^. In contrast, Type B particles were emitted from Unit 1 and are larger than Type A particles, mainly non-spherical particles. The ^134^Cs/^137^Cs radioactivity ratio of the inventory in Unit 1 is estimated to be 0.94^11^. These studies collected CsMPs from various environmental samples, including aerosol filters, soils, plants, and river sediments.

CsMPs were transferred not only around the FDNPP but also over a wide area of the Kanto region. The Kanto region is a geographical area of Honshu, Japan's largest island, and includes Tokyo and six neighboring prefectures. Ikehara et al.^5^ reported the plume trajectories of material released from the FDNPP on March 14, 2011, in the late afternoon through to the late afternoon of March 15, 2011, indicating that the CsMPs were formed only during this short period. Tsuruta et al.^12^ reported that the mainstream plume containing radiocesium at the time of the accident occurred in nine stages, with plumes containing CsMPs existing from the first stage on March 12, 2011, to the fourth stage on March 16, 2011. The plumes after the fifth stage did not reach the Kanto region or had no CsMP present^12–16^. Utsunomiya et al.^4^ and Abe et al.^17^ isolated some CsMPs from aerosol filter samples in the Kanto region and neighboring prefectures. Higaki^7^ also reported that 22 CsMPs were successfully isolated from a face mask worn on March 15–16, 2011, in Tokyo metropolitan. These CsMPs transferred to the Kanto region were found to have originated from Unit 2 based on their shapes, elemental compositions, and ^134^Cs/^137^Cs radioactivity ratios. Some studies have suggested the possibility of the resuspension of CsMPs that have settled on the ground^18,19^. CsMPs are extremely small particles and are easily taken up into the body by inhalation; they have the potential to remain in organs such as the lungs for a long time. In such cases, they might cause higher internal exposure than that caused by the same radioactivity of soluble radiocesium because of the long retention time in the body. Several studies have been conducted to investigate the dissolution behavior of CsMPs in simulated body fluids^20,21^.

Since CsMPs were discovered, their properties and environmental fates have been studied using various analytical tools. However, there are few reports on CsMPs that have not been affected by rainfall in the Kanto region, the most populous region in Japan^4,7,17^. The CsMPs found in these studies were collected from the air before they fell to the ground, while most CsMPs were believed to have fallen to the ground before collection. It cannot be ruled out that CsMPs with new characteristics not previously reported may exist in the Kanto region, other than the typical spherical CsMPs that originated from Unit 2. Therefore, this study aimed to analyze samples collected from the dry deposition of fallout from the plume that arrived in the Kanto region immediately after the accident and to investigate the characteristics of CsMPs. The novel contribution of this study is that the dry deposition samples were collected from the plume fallout over a large area of the Kanto region before the first post-accident rainfall.

## Materials and methods

### Sample collection

**Figure 1:**
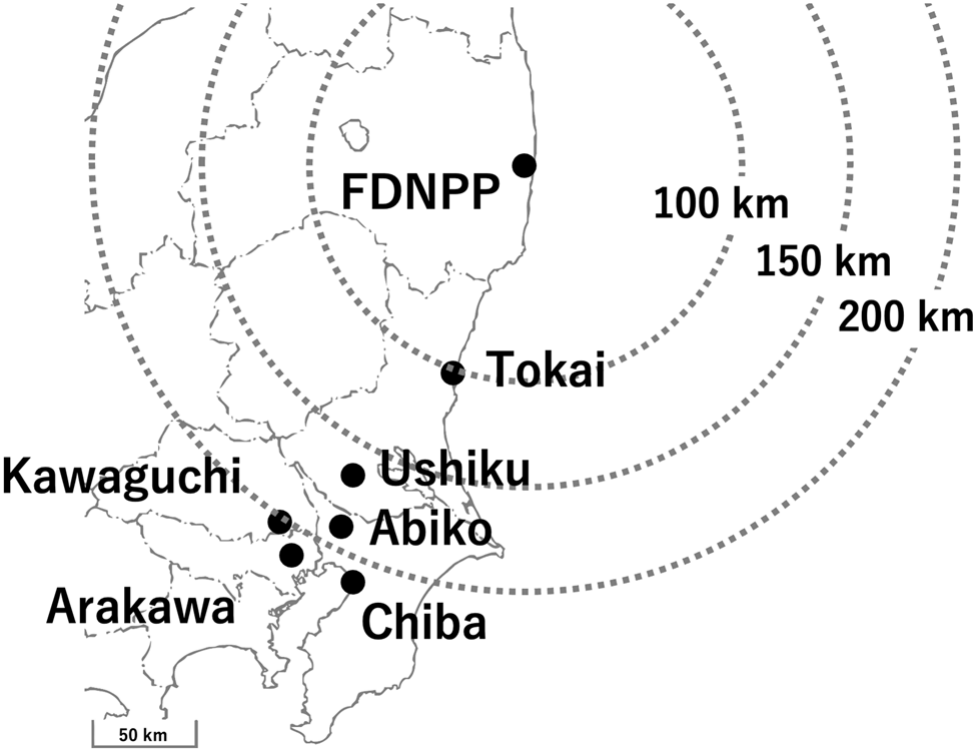
Sampling locations for this study, created using Adobe Illustrator software (version 28.0) and Microsoft PowerPoint (version 16.79). Circles indicate distances from the FDNPP.

Sampling was conducted using a wiping method similar to that used in surface contamination surveys at radiation facilities. Flat and smooth surfaces such as metal handrails on balconies and car hoods were lightly wiped with tissue papers with an area of approximately 100 cm^2^. Those smooth outdoor surfaces were targeted for sampling because dry fallout deposition was assumed to result in easy-to-remove contamination on these surfaces immediately after the accident. Figure 1 shows each sampling location and its approximate distance from the FDNPP. Four samples were collected from Tokai village on March 20, 2011. The first three samples were collected from different locations, whereas the fourth sample was collected by wiping the same location from which the third sample had been collected. Three samples were collected from car hoods in Ushiku on March 17, 18, and 20. From Kawaguchi City, one sample was collected on March 16, and two samples were collected on March 20. One sample each was collected from Arakawa Ward on March 17, Abiko City on March 21, and Chiba City on March 21. The first rainfall observed in these areas after the accident was recorded on March 21, 2011, which allowed us to sample dry deposition that was not washed out by rain. As each location was on private property, sample collection was performed by different residents in each case. Each tissue paper sample was placed in a zipper bag to avoid cross-contamination. The samples were stored in the dark until analysis in 2019. The presence of CsMPs was not evident at the time of sampling. Therefore, the tissue paper samples were initially intended only for monitoring the surface contamination caused by dry fallout deposition. Then, in 2013, the discovery of CsMPs^2^ and the establishment of the wet separation method^7,8,22^ enabled many CsMPs to be easily isolated. This collecting method was difficult to apply after rain because dry deposition contamination on smooth surfaces outdoors decreases over time. The isolation of CsMPs from the stored tissue paper samples was performed in 2019.

### Radioactivity measurement of tissue paper samples

Each tissue paper sample was packed into a cylindrical plastic vessel (47 mm diameter, 6 cm height) and analyzed using a high-resolution gamma spectrometry system with a high-purity germanium detector (GX4018, Mirion Technologies (Canberra) Inc.). However, two samples collected from Kawaguchi City on March 20 were packed in a cylindrical plastic vessel as one measurement sample. The counting time for each sample was 21,600–50,000 s. Spectrum Explorer software (Mirion Technologies (Canberra) Inc.) was used to calculate the radioactivity (Bq) of ^134^Cs and ^137^Cs at the time of the accident (March 11, 2011). A standard radioactivity gamma-volume source (MX033U8PP, Japan Radioisotope Association) was used to determine the counting efficiency of the gamma spectrometry system. The standard radioactivity source was calibrated using the Japan Calibration Service System. Self-absorption correction for ^134^Cs and ^137^Cs and coincident counting correction for ^134^Cs were performed by a function of Spectrum Explorer software according to the method described in the series of environmental radioactivity measuring methods No. 7^23^. Here, the tissue paper samples in each plastic vessel were considered homogeneous in the gamma-ray analysis. However, in reality, the CsMPs were heterogeneously distributed in the sample cases because only a few CsMPs were collected on each tissue paper sample. Therefore, the activity values measured by the Ge detector could have greater uncertainty than individual counting errors.

### Estimation of wiping efficiency

The Tokai-3 and Tokai-4 samples were wiped from the same location using separate tissue papers. In general, wiping efficiency is calculated by comparing the radioactivity of the easy-to-remove contaminant present before wiping with the radioactivity of the wiped sample. The formula for determining the wiping efficiency is as follows:

1 $$E = A_{w} /A$$

where *E* is the wiping efficiency, *A*_*w*_ is the wiped radioactivity, and *A* is the radioactivity of the easy-to-remove surface contamination.

### Isolation of CsMPs

**Figure 2:**
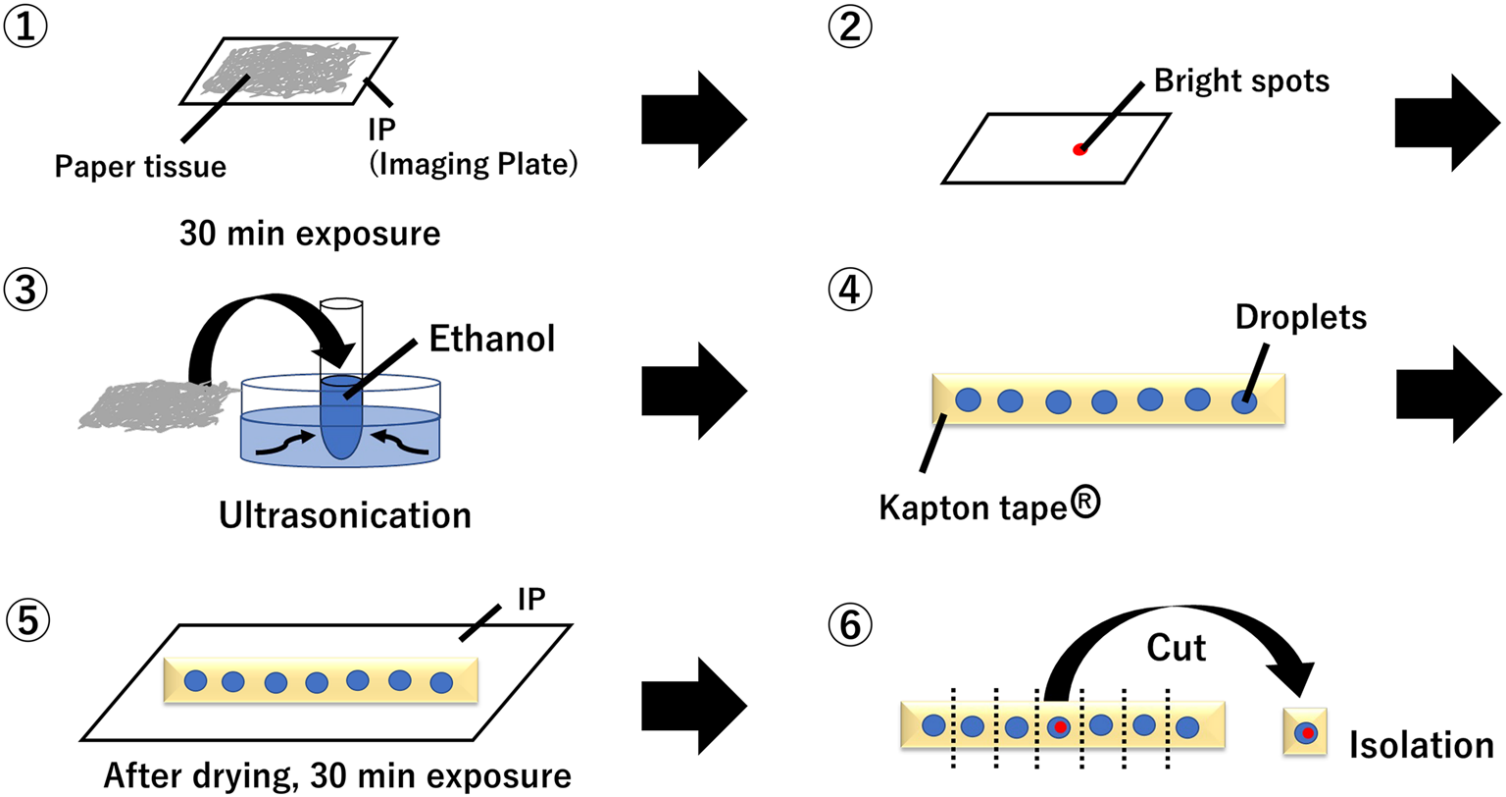
Flow of the wet method used to isolate CsMPs.

The wet method was used to isolate CsMPs from samples collected using the wiping method. The method was modified as described in previous studies^7,8,22^. Figure 2 shows the flow of the wet method used to isolate the CsMPs. (1) Tissue paper samples were placed on an imaging plate (IP) (BAS-MS2040, FujiFilm) and exposed for 30 min. The tissue paper samples and IP were set into a cassette (BAS Cassette 2040, FujiFilm) during exposure to avoid light-induced fading. (2) The plate was read with an IP reader (FLA-9000, FujiFilm) to confirm the presence of CsMPs on the tissue. (3) CsMPs were isolated from the tissue paper samples as follows. A square area of approximately 2 mm surrounding the high-radioactivity spots in the tissue paper was cut. The fragment of the tissue paper was placed in a 3 mL plastic tube. Subsequently, ethanol (1 mL) was added to the tube. The tube was sonicated in an ultrasonic washer bath (AS482, AS ONE Corporation) for 15 min to transfer the CsMPs from the fragment to the solution. (4) A drop of the ethanol solution used to ultrasonically sonicate the tissue paper was dropped on a piece of Kapton® tape. (5) After drying, the Kapton® tape was placed on the IP and exposed for 30 min, and the plate was read with the IP reader. (6) CsMPs were isolated by cutting out the Kapton® tape corresponding to the location where the bright spots were identified. The CsMPs were named by “sampling location” and “sampling date (year, month and day)” followed by alphabetical letters in the order of which they were found.

Based on previous findings^23^, high-radioactivity spots indicated the presence of CsMPs. Because of the short time (20–30 min) of exposure to the IP, only the points where radiocesium was accumulated at high concentrations could be visualized. The radioactivity of radiocesium absorbed on clay particles is low owing to the original weight of the clay particles. Therefore, if only these clay particles exist, because of the short exposure time to IP, we may not be able to visualize the particles. Higaki et al.^24^ reported that the face mask sample that was exposed to the IP for 144 h showed several bright spots. Upon microscopic observation of the locations of these bright spots, we found that all spots were associated with soil particles containing radiocesium. Furthermore, in the wet method, soluble particles were dissolved, leaving behind only insoluble particles. These viewpoints are supported by previous studies^7,8^. In this study, even some samples with bright spots showed no significant ^137^Cs radioactivity by gamma-ray measurements using the Ge detector, and these were excluded. The lowest CsMP radioactivity value we observed with this method was 0.017 Bq for ^137^Cs (Mask-284-D in Higaki et al.^8^). We believe that this value is close to the detection limit of the method.

During step (2) of analysis of the Ushiku-20110320 tissue paper sample, a large asymmetric bright spot was observed on the IP (Supplementary Fig. S1). As the asymmetric spot was larger than typical spherical CsMPs, we assumed that high concentrations of soluble radiocesium particles may be present. Therefore, for only this particle, we decided not to proceed with wet separation after step (3) and instead attempted a dry separation method. The dry separation method was modified as described in a previous study^9^. The fragment was placed on a Kapton® tape. To analyze the high-radioactivity spot, we attempted to remove fiber from the tissue paper by repeatedly stacking and peeling off another Kapton® tape. The Kapton® tape was placed on an IP and exposed for 30 min to confirm the presence of the CsMPs. The particle observed was named Ushiku-20110320-B, following the individual naming rules of CsMPs in this study. Here, the bright spot was split into eight pieces using the dry separation method; it was revealed that Ushiku-20110320-B was easily broken. Therefore, the gamma-ray measurement (as described in the next section) of Ushiku-20110320-B was performed before complete isolation.

### Analysis of CsMPs

The gamma rays of ^134^Cs and ^137^Cs were measured using either of the Ge semiconductor detectors (NIGC2020, Princeton Gamma-Tech Instruments Inc. and GX4018, Mirion Technologies (Canberra) Inc.) for 500,000–600,000 s. The CsMPs on a fragment of Kapton® tape were placed at the center of a small plastic case (38 × 66 mm, 13 mm height, and 2 mm bottom thickness). The detection limits of the detectors for ^134^Cs and ^137^Cs were 0.00577 Bq and 0.00537 Bq, respectively, each with a counting time of 600,000 s. To determine the ^134^Cs/^137^Cs radioactivity ratio of all CsMPs collected from each tissue paper sample with high accuracy, all CsMPs on the Kapton® tape fragments collected from each tissue paper sample were set on one small plastic case as one measurement sample. A CsMP, named “Mask-12-A,” found in a mask in a previous study^7^ was used as a radioactivity standard. The radioactivity was 4.68 ± 0.11 Bq for ^134^Cs and 25.3 ± 0.1 Bq for ^137^Cs as of December 2, 2016, which was determined by comparing a 1-mm square filter paper containing radioactivity standard solutions of ^134^Cs (CZ-010, Japan Radioisotope Association) and ^137^Cs (CS-005, Japan Radioisotope Association). These standard radioactivity solutions were calibrated to three significant digits using the Japan Calibration Service System. To obtain more accurate ^134^Cs/^137^Cs values, we prepared composite measurement samples at each sampling location. Each Kapton® tape fragment was set on one small plastic case as a composite measurement sample. The composite samples were named by “sampling location” and “sampling date (year, month and day)” followed by all_CsMPs. The radioactivity was measured using the high-resolution gamma spectrometry systems for 150,000–400,000 s. Furthermore, the radioactivity of the composite measurement samples was determined using a standard CsMP named “Mask-12-A,” in which only one CsMP particle was placed in the center of a single plastic case. Therefore, there is a possibility that the total radioactivity was underestimated. Details of the quantitative measurements of CsMPs were provided in previous studies^3,7,8^.

Some isolated CsMPs were observed using scanning electron microscopy-energy dispersive X-ray spectrometry (SEM–EDS) (SEM: S-4500, Hitachi, EDS: Sigma, Kevex Instruments). The acceleration voltage was 15 kV, the emission current was 15 µA, the working distance was 12 mm, and the tilt was 15°. Radioactive mineral thorianite was used as a marker to facilitate SEM observations. Carbon coating was conducted before SEM observation (SC-701C, Sanyu Electron).

### Trital re-isolation of CsMPs from Kapton® tape fragments

The wet separation method using ethanol applied in this study has the advantage of reducing drying time. However, CsMP can enter the adhesive of the Kapton® tape. If a CsMP is embedded in this adhesive, SEM observation becomes impossible. Therefore, we tried to re-isolate the CsMPs named Ushiku-20110320-A and C and one of the eight pieces of Ushiku-20110320-B by removing the adhesive material using a wet method using acetone (FUJIFILM Wako Pure Chemical Corporation, Guaranteed Reagent) and *N*,*N*-dimethylformamide (DMF) solution (FUJIFILM Wako Pure Chemical Corporation, Guaranteed Reagent) before SEM observation. First, 21 ultrasonic cleaning cycles were performed using acetone. Then, the 22nd cycles and subsequent ultrasonic cleaning was performed using DMF. The re-isolation trial was conducted in 2022, 11 years after the accident. As shown in Supplementary Fig. S2, ultrasonication was then performed for 1 h. The Kapton® tape fragment was removed from the tube. After drying, it was placed on an IP and exposed for 20 min. If no distinct spots were identified, we assumed that the CsMPs were removed from the Kapton® tape fragment.

## Results

### Analysis of CsMPs dispersed over a wide area in the Kanto region

In this study, 106 CsMPs were successfully isolated from tissue samples collected at six sampling sites. Table 1 lists the CsMPs found in this study with activities of ^134^Cs and ^137^Cs exceeding 0.50 Bq at the time of the accident (March 11, 2011) (details of all CsMPs are shown in Supplementary Table S1). Sampling locations and the number of CsMPs found in the tissue paper samples are shown in Supplementary Fig. S3. Among the sampling sites, CsMPs were found in all tissue paper samples collected in this study. As described in the Materials and Methods section, the gamma ray measurement of Ushiku-20110320-B was performed before complete isolation. As the Kapton® tape fragment may have also contained soluble radiocesium that would have been dissolved and removed by the wet method, the radioactivity of Ushiku-20110320-B might be overestimated.

**Table 1:** CsMPs found in this study with ^134^Cs and ^137^Cs exceeding 0.50 Bq at the time of the accident. The measurement error shows 1σ.

Sample	At the accident		
	^134^Cs (Bq)	^137^Cs (Bq)	^134^Cs/^137^Cs
Tokai1-20110320-G	0.734 ± 0.196	0.691 ± 0.007	1.06 ± 0.28
Tokai1-20110320-N	0.829 ± 0.196	0.682 ± 0.007	1.22 ± 0.29
Tokai1-20110320-O	1.24 ± 0.26	1.09 ± 0.01	1.14 ± 0.23
Tokai1-20110320-U	0.929 ± 0.172	0.855 ± 0.007	1.09 ± 0.20
Tokai2-20110320-A	0.607 ± 0.182	0.655 ± 0.006	0.927 ± 0.277
Tokai2-20110320-B	1.01 ± 0.31	0.717 ± 0.007	1.41 ± 0.43
Tokai2-20110320-C	1.50 ± 0.26	1.30 ± 0.01	1.15 ± 0.19
Tokai2-20110320-E	2.62 ± 0.33	2.41 ± 0.01	1.09 ± 0.14
Tokai2-20110320-G	0.562 ± 0.169	0.538 ± 0.005	1.04 ± 0.31
Tokai3-20110320-C	2.06 ± 0.22	1.78 ± 0.01	1.16 ± 0.12
Tokai3-20110320-D	0.648 ± 0.232	0.573 ± 0.007	1.13 ± 0.41
Tokai3-20110320-F	0.798 ± 0.190	0.814 ± 0.006	0.980 ± 0.234
Tokai3-20110320-H	0.724 ± 0.259	0.551 ± 0.008	1.31 ± 0.47
Tokai3-20110320-J	3.41 ± 0.31	3.14 ± 0.02	1.09 ± 0.10
Tokai3-20110320-K	0.729 ± 0.205	0.602 ± 0.007	1.21 ± 0.34
Tokai3-20110320-N	1.25 ± 0.33	1.06 ± 0.01	1.32 ± 0.47
Tokai4-20110320-A	0.674 ± 0.164	0.699 ± 0.006	0.965 ± 0.235
Tokai4-20110320-C	1.48 ± 0.26	1.25 ± 0.01	1.19 ± 0.21
Ushiku-20110318-A	1.17 ± 0.20	1.05 ± 0.01	1.11 ± 0.19
Ushiku-20110320-A	2.15 ± 0.23	2.01 ± 0.01	1.07 ± 0.11
Ushiku-20110320-B	6.59 ± 0.54	5.81 ± 0.03	1.13 ± 0.09
Ushiku-20110320-C	0.904 ± 0.128	0.788 ± 0.007	1.15 ± 0.16
Arakawa-20110317-A	0.872 ± 0.140	0.828 ± 0.006	1.05 ± 0.17
Arakawa-20110317-B	0.803 ± 0.143	0.800 ± 0.006	1.00 ± 0.18
Arakawa-20110317-G	0.559 ± 0.121	0.567 ± 0.005	0.986 ± 0.214
Arakawa-20110317-J	0.779 ± 0.127	0.621 ± 0.006	1.26 ± 0.21
Arakawa-20110317-L	0.916 ± 0.165	0.765 ± 0.008	1.20 ± 0.22
Abiko-20110321-B	0.694 ± 0.169	0.629 ± 0.007	1.10 ± 0.27
Abiko-20110321-C	1.88 ± 0.34	1.74 ± 0.01	1.08 ± 0.19
Kawaguchi-20110316-G	0.721 ± 0.183	0.649 ± 0.007	1.11 ± 0.28
Kawaguchi1-20110320-D	0.546 ± 0.330	0.539 ± 0.007	1.01 ± 0.61

### Estimation of ^134^Cs/^137^Cs radioactivity ratios by γ-ray measurement

By clarifying the ^134^Cs/^137^Cs radioactivity ratio and the elemental compositions of the CsMPs, which were decay-corrected to the time of the accident, the types of CsMPs could be identified. However, because more than 10 years had elapsed between sample collection and radioactivity measurements, there were 43 samples in which ^134^Cs, which has a short half-life of 2.0648 y, was not detected. From the radioactivity measurement results, ^134^Cs/^137^Cs radioactivity ratios were calculated. Table 2 lists the radioactivity ratios of the CsMPs collectively measured at each sampling point to the total radiocesium wiped onto the tissue paper samples. At most sampling locations, the ^134^Cs/^137^Cs radioactivity ratio was close to 1.08 which was similar to that in the inventory of FDNPP Unit 2 calculated with the ORIGEN code by Nishihara et al.^11^. However, because of measurement errors in the ^134^Cs/^137^Cs radioactivity ratios, the origin of these CsMPs could not be determined from this value alone.

**Table 2:** Radioactivity of ^134^Cs and ^137^Cs and the radioactivity ratio of composite measurement samples at each sampling point. The CsMP ratio shows the radioactivity of CsMPs to the total radiocesium wiped on the tissue paper sample. The measurement error shows 1σ.

Sample	At the accident			CsMP Ratio (%)
	^134^Cs (Bq)	^137^Cs (Bq)	^134^Cs/^137^Cs	
Tokai1-20110320-all_CsMPs	8.30 ± 0.59	7.07 ± 0.03	1.17 ± 0.17	
Tokai1-20110320-tissue_paper	24.0 ± 0.8	21.8 ± 0.2	1.10 ± 0.17	33.6
Tokai2-20110320-all_CsMPs	9.88 ± 0.91	8.99 ± 0.05	1.10 ± 0.10	
Tokai2-20110320-tissue_paper	25.7 ± 1.1	24.3 ± 0.2	1.06 ± 0.11	37.8
Tokai3-20110320-all_CsMPs	11.2 ± 0.9	10.1 ± 0.1	1.10 ± 0.09	
Tokai3-20110320-tissue_paper	24.9 ± 0.9	22.5 ± 0.2	1.10 ± 0.09	45.0
Tokai4-20110320-all_CsMPs	2.84 ± 0.31	2.48 ± 0.02	1.15 ± 0.13	
Tokai4-20110320-tissue_paper	11.5 ± 0.6	11.5 ± 0.2	1.00 ± 0.14	23.2
Ushiku-20110317-all_CsMPs	0.517 ± 0.116	0.461 ± 0.006	1.12 ± 0.25	
Ushiku-20110317-tissue_paper	7.22 ± 0.43	6.19 ± 0.11	1.17 ± 0.26	7.30
Ushiku-20110318-all_CsMPs	2.13 ± 0.25	2.09 ± 0.01	1.02 ± 0.12	
Ushiku-20110318-tissue_paper	7.19 ± 0.45	7.13 ± 0.10	1.01 ± 0.14	29.4
Ushiku-20110320-all_CsMPs	11.2 ± 0.7	9.83 ± 0.04	1.14 ± 0.19	
Ushiku-20110320-tissue_paper	23.4 ± 0.8	21.7 ± 0.2	1.08 ± 0.20	46.6
Abiko-20110321-all_CsMPs	3.31 ± 0.38	2.91 ± 0.02	1.14 ± 0.13	
Abiko-20110321-tissue_paper	16.9 ± 0.6	16.6 ± 1.4	1.02 ± 0.17	18.6
Chiba-20110321-all_CsMPs	0.278 ± 0.114	0.274 ± 0.005	1.02 ± 0.42	
Chiba-20110321-tissue_paper	2.73 ± 0.22	2.78 ± 0.05	0.980 ± 0.359	10.0
Kawaguchi-20110316-all_CsMPs	3.17 ± 0.36	2.64 ± 0.02	1.20 ± 0.14	
Kawaguchi-20110316-tissue_paper	12.6 ± 0.6	12.1 ± 0.1	1.04 ± 0.15	23.5
Kawaguchi-20110320-all_CsMPs_1 + 2	2.00 ± 0.21	1.72 ± 0.01	1.17 ± 0.34	
Kawaguchi-20110320-tissue_paper_1 + 2	6.20 ± 0.43	5.66 ± 0.09	1.10 ± 0.35	31.4
Arakawa-20110317-all_CsMPs	6.00 ± 0.44	5.33 ± 0.03	1.12 ± 0.08	
Arakawa-20110317-tissue_paper	13.5 ± 0.6	12.9 ± 0.1	1.04 ± 0.10	42.9

The total radioactivity of the composite measurement samples might be underestimated, and the radioactivity of the tissue paper samples could have greater uncertainty than individual counting errors.

As shown in Table 2, the radioactivity ratio of CsMPs to the total radiocesium in samples collected at each sampling location was calculated and found to be 7.3–46.6%. As described in the Materials and Methods section, there is a possibility that the total radioactivity of the composite sample was underestimated. Therefore, the radioactivity ratios of CsMPs to the total radiocesium might be even higher.

### Analysis of CsMPs via SEM–EDS

**Figure 3:**
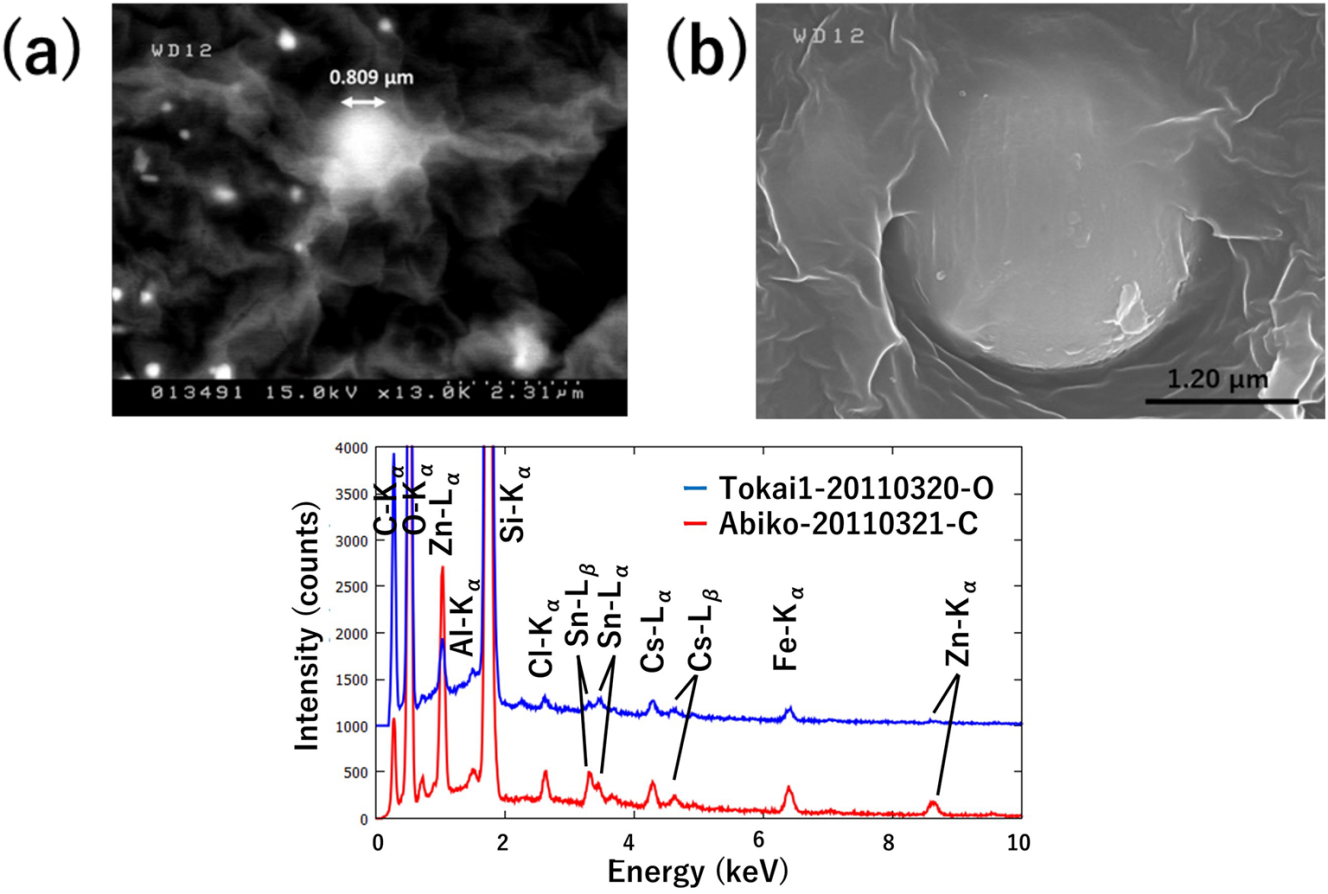
SEM images and EDS spectra of CsMPs. (**a**) Tokai1-20110320-O, (**b**) Abiko-20110321-C.

SEM–EDS analysis was performed on 19 samples with radioactivity of ^137^Cs above 0.50 Bq by γ-ray measurement. Consequently, two CsMPs were successfully observed, and elemental analysis was conducted. Figure 3 shows SEM images of CsMPs and the EDS spectra of Tokai1-20,110,320-O and Abiko-20110321-C. The remaining CsMPs were buried in Kapton® tape and could not be analyzed. The SEM image of Tokai1-20110320-O shows that it is spherical with a diameter of 0.809 µm. The SEM image of Abiko-20110321-C showed that the particles were spherical with a diameter of 2.2 µm. These indicated Type A characteristics. The EDS analysis also confirmed the presence of O, Si, Cl, Al, Fe, Zn, Sn and Cs, which are the main constituents of typical Type A CsMPs.

### Analysis of two particles found in Ushiku City

**Figure 4:**
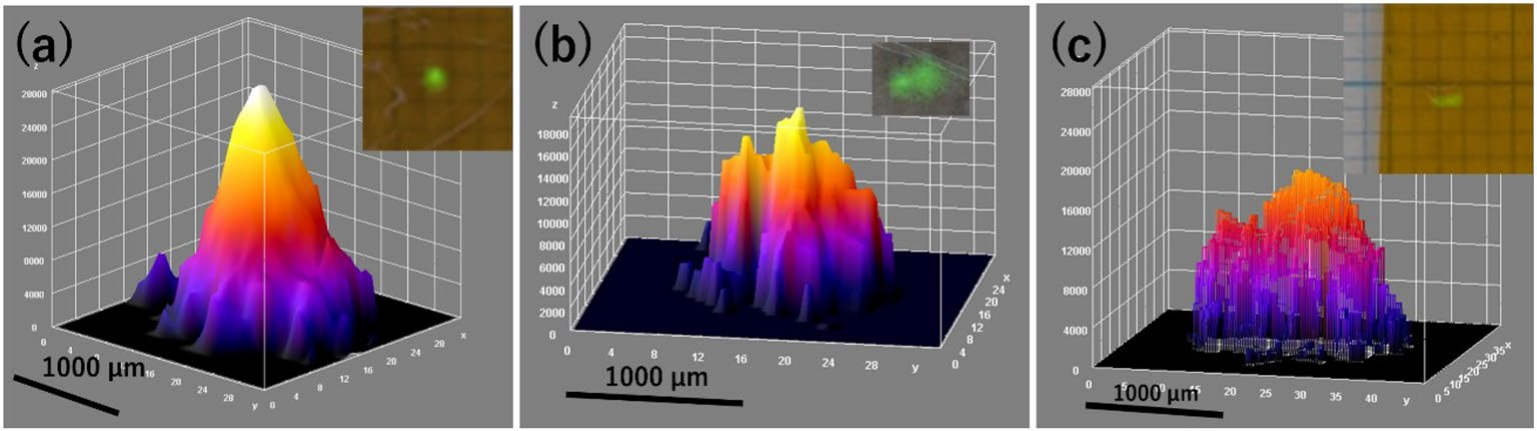
Surface distribution images of CsMPs found in this study using ImageJ software. (**a**) Ushiku-20110320-A (spherical), (**b**) Ushiku-20110320-B, (**c**) Ushiku-20110320-C.

The two asymmetric bright spots on the IP images of CsMPs (Ushiku-20110320-B and C) found in Ushiku, Ibaraki Prefecture, Japan, suggest the possibility of the presence of non-spherical CsMPs or the presence of two spherical Type A CsMPs considered to be stuck together. Additionally, they were compared with Ushiku-20110320-A, a spherical Type A particle. Figure 4 shows the surface distribution images of spherical and non-spherical CsMPs analyzed using ImageJ software^25,26^. Radioactivity measurements indicated that Ushiku-20110320-B and C were Type A, as the ^134^Cs/^137^Cs values were 1.13 ± 0.09 and 1.15 ± 0.16, respectively (Table 1).

For Ushiku-20110320-B, we conducted a dry separation method. When the Kapton® tape was stuck and peeled off, bright spots were separately observed on the IP. This suggests that the separated bright spots are radioactive materials derived from the CsMPs that were broken up by the adhesive.

### Re-isolation of CsMPs from Kapton® tape fragments

**Figure 5:**
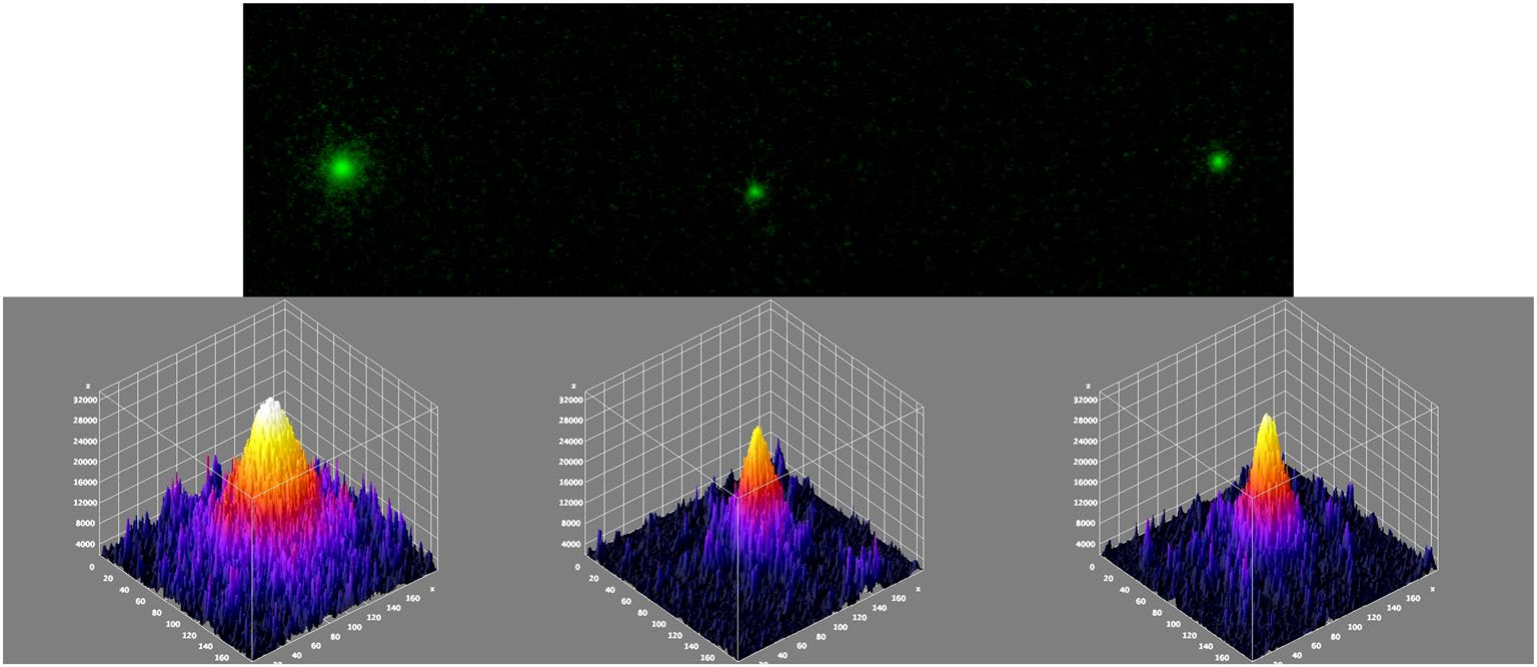
Bright spots of CsMPs after 30 min exposure on an IP with surface distribution images obtained using ImageJ software. (Left) Non-spherical CsMP named “Mask-12-A” found in a mask in a previous study^3^. (Center) Spherical CsMP named “Tokai1-20110320-O” found in this study. (Right) Spherical CsMP named “Abiko-20110321-C” found in this study.

SEM–EDS analysis of these CsMPs called Ushiku-20110320-A and C and one of the eight pieces of Ushiku-20110320-B could not be performed because they were buried in the Kapton® tape adhesive. We attempted to re-isolate them by the wet method using acetone and DMF solution; however, they could not be isolated because the bright spots still remained. Figure 5 shows changes in the bright spots after re-isolation experiments with a 3D surface plot. Surface distribution analysis using ImageJ software showed that the intensity and distribution of bright spots decreased after each re-isolation experiment for Ushiku-20110320-B and C. In contrast, almost no change was observed for Ushiku-20110320-A.

## Discussion

In this study, CsMPs deposited on the ground in outdoor living areas were collected using the wiping method, which has never been reported before, and isolated using the wet method. Using this method, 106 CsMPs were successfully isolated from the six sampling sites. Based on the calculated ^134^Cs/^137^Cs radioactivity ratios, the CsMPs that were deposited on the ground over a wide area of the Kanto region were most likely Type A particles. SEM–EDS analysis of the successfully separated CsMPs showed that the particles were spherical in shape and contained Si, Cs, Fe, Zn, Sn, and Na, which are the major components of Type A particles^27,28^. Abe et al.^17^ reported that the CsMPs that reached the Kanto region were derived from Unit 2. The plume and CsMPs that arrived at Hongo campus of the University of Tokyo on March 15–16, 2011, from the FDNPP were also from Unit 2^7,29^. Therefore, the CsMPs identified in this study were derived from Unit 2. Notably, CsMPs with a ^134^Cs/^137^Cs radioactivity ratio below 1.00 were also found; however, this could be caused by large measurement errors owing to the small counting values of ^134^Cs, which was a key factor in identifying the CsMP type, as approximately 10 years had passed from the sampling date to the radioactivity measurement date (half-life of ^134^Cs: 2.0648 y).

The plume containing CsMPs arrive in the Kanto region on March 15, 2011. Some of the radiocesium including CsMPs, was immediately deposited on the ground on the same day and was unaffected by rain. Many of the CsMPs remained in the same location until the latest sampling date in this study (March 21, 2011). In contrast, other forms of radiocesium excluding CsMPs may have been scattered by wind, which may have increased the CsMP to total radiocesium radioactivity ratios. Alternatively, the method used in this study may have been more efficient for wiped CsMPs but less efficient for detecting other forms of radiocesium. This sampling was conducted amid the chaos following the Great East Japan Earthquake and the FDNPP accident, and daily life had not yet completely returned to normal. Ideally, established methods such as using smearing paper to determine wiping efficiency should have been used in this study. We attempted to estimate the wiping efficiency in this study using the Tokai-3 and Tokai-4 data. Using the result of radioactivity of the first wipe (Tokai-3), we get

2 $$E = 24.9/A$$

and from the result of radioactivity of the second wipe (Tokai-4), we get

3 $$E = 11.5/\left( {A - 24.9} \right)$$

The wiping efficiency was assumed to be constant for the first and second wiping. The simultaneous equations (Eqs. (2) and (3)) were solved for *A* and* E*.

4 $$24.9/A = 11.5/\left( {A - 24.9} \right)$$

Therefore, *A* = 46.3 Bq. We substituted the solution for *A* into Eq. (2). The wiping efficiency was estimated to be *E* = 24.9/46.3 = 53.8%. Therefore, the wiping efficiency was 53.8%, which is not significantly different from the standard value of 50% used in ISO 7503–2:2016 ^30^ for smooth metal surfaces. Therefore, the methodology of this study is appropriate and in accordance with standard monitoring methods at radiation facilities. However, it is likely that not all deposited radiocesium was removed. Additionally, there may have been individual differences in sampling as different people performed sampling at each location. It may be necessary to define the collection efficiency of each particle, such as CsMPs collected from tissue paper, to evaluate the CsMP ratio in the plume more accurately. According to airborne monitoring by MEXT of Japan and the U.S. Department of Energy, (May 18–26, 2011)^31^, the total ^134^Cs + ^137^Cs of the deposition in Tokai village was under 100 kBq/m^3^. Thus, the values obtained in this study (46.3 Bq/100 cm^2^ i.e. 4.63 kBq/m^3^) can be evaluated as reasonable.

In this study, the radioactivity ratio of CsMPs to the total radiocesium in the samples collected at each sampling location was 7.3–46.6%. Some other studies showed the ratio in the plume collected immediately after the accident in the Kanto region. Utsunomiya et al.^4^ estimated the ratio to be 80.8–88.8% by dissolution of air sample filters. Higaki^7^ showed that CsMP to total radiocesium radioactivity ratio was 9.9% which was collected on a face mask worn outdoor on March 15–16, 2011; it is possible that CsMPs with a diameter below 1.89 µm were excluded. Higaki et al.^3,8^ showed that the maximum ratio was approximately 4% in samples collected from face masks worn during the indoor cleaning of residences in the evacuation area near the FDNPP. Fueda et al.^6^ showed that the CsMP to total radiocesium radioactivity ratios in indoor dust at an elementary school in Okuma Town located 2.8 km south-west of the FDNPP was 4.48–38.9%. Compared to the values in these studies, the values in the present study were closer to those of Fueda et al.^6^ However, as mentioned above, tissue paper sampling might not be considered uniform sampling for soluble radiocesium or CsMPs. Furthermore, the proportion of CsMPs in the plume might not be uniform.

The bright spots on the IPs were not spherical, suggesting that the two CsMPs found in Ushiku City may have non-spherical shapes. Figure 4 compares radiographic images of typical shapes and irregular shapes on the IP found in this study. Although non-spherical Type A CsMPs have been identified near the FDNPP in Fukushima prefecture^3,10,32–37^, their discovery has not been reported in the Kanto region. We calculated the ^134^Cs/^137^Cs ratio and found that the obtained values of Ushiku-20110320-B and C were closest to the value derived from Unit 2, suggesting that they are Type A particles. Strictly speaking, it was not possible to demonstrate that the values measured were for Unit 2 and different from the ORIGEN code values for Units 1 and 3 within the limits of the measurement error. If bright spots with non-spherical irregular shapes are observed on an IP (Fig. 4b and c), they could be considered two spherical Type A CsMPs stuck together. Therefore, a non-spherical CsMP, named “Mask-12-A,” found in a mask in a previous study^3^ and spherical CsMPs “Tokai1-20110320-O” and “Abiko-20110321-C” found in this study were also exposed to IP for 30 min. As shown in Fig. 5, not only the spherical CsMPs but also the non-spherical CsMPs showed spherical bright spots. Therefore, we concluded that Ushiku-20110320-B and Ushiku-20110321-C were two spherical Type A CsMPs stuck together.

**Figure 6:**
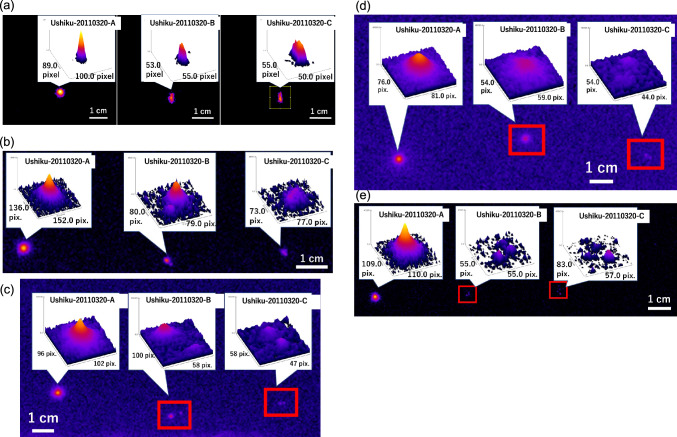
Changes in bright spots from re-isolation experiments of spherical CsMP (Ushiku-20110320-A) and CsMPs suggested to be non-spherical (one of eight pieces of Ushiku-20110320-B and Ushiku-20110320-C) with a 3D surface plot. (**a**) IP of the samples sensitized for 24 h by performing the WET method with acetone seven times. (**b**) IP of the samples sensitized for 24 h by performing the WET method with acetone 17 times. (**c**) IP of the samples sensitized for 48 h by performing the WET method with acetone 21 times. (**d**) IP of the samples sensitized for 48 h by performing the WET method with DMF 34 times. (**e**) IP of the samples sensitized for 24 h by performing the WET method with DMF 37 times.

We tried to observe the particles Ushiku-20110320-B and C and performed elemental analysis using SEM–EDS. However, we were unable to observe them because they were buried in the tape adhesive. We tried to re-isolate the particles by the wet method using acetone and DMF and found that the shape, intensity, and distribution of the bright spots changed even though they remained on the tape. Figure 6 shows changes in the bright spots from re-isolation experiments of Ushiku-20110320-A (a spherical CsMP) and Ushiku-20110320-B and C. No change was observed in Ushiku-20110320-A, which was subjected to the same re-isolation experiment. Thus, it is estimated that Ushiku-20110320-B and C were brittle CsMPs and they dissolved gradually. Additionally, Higaki et al.^8^ reported the presence of high concentrations of soluble radiocesium particles or aerosols adhered to house dust because of a broad asymmetric distribution of bright spots after dissolving the samples only twice using ultrasonication in methanol. In contrast, the bright spots of Ushiku-20110320-B and -C were reduced by the wet method using acetone and DMF solution in this study. Nevertheless, the bright spots remained after ultrasonication performed 37 times. These results imply that the main origin of the bright spots were CsMPs, not high concentrations of soluble radiocesium particles or aerosols.

Additionally, dry separation experiments using Kapton® tape revealed that the CsMP called Ushiku-20110320-B easily disintegrated, even in the absence of weathering effects. It is believed that the reason that the CsMPs have become more fragile 10 years after the accident is the large specific surface area of non-spherical particles, which were physically broken by the ultrasonic processing conducted in this study. The following two factors can be considered as causes of CsMPs becoming more fragile over time: (1) an imbalance at the atomic level within the particles because of the conversion of ^134^Cs and ^137^Cs to ^134^Ba and ^137^Ba, respectively, via radioactive decay, and (2) damage from beta and gamma rays accompanying the decay of ^134^Cs and ^137^Cs.

Within the particles, Cs is in the chemical form of CsAlSiO_4_ or Cs_2_SiO_3_^38^, and radiogenic transformation from Cs to Ba may result in the imbalance of the elemental arrangement and composition within the particles, leading to their fragility. In particular, ^134^Cs may be a key factor because of its short half-life of 2.0648 years. According to Kogure et al.^27^, a typical Type A CsMP has approximately 10 wt% Cs in the particles. Nishihara et al.^11^ calculated the weight of Cs isotopes at the accident and found the weights of the FDNPP Unit 2 core as follows: 76.4 kg for ^133^Cs, 5.78 kg for ^134^Cs, 26.7 kg for ^135^Cs, and 79.5 kg for ^137^Cs. In this study, the separation experiments were conducted 10 years after the accident, and we determined how much Cs was converted to Ba. As ^133^Cs is a stable isotope and the half-life of ^135^Cs is 1.33 × 10^6^ y, the amount of these isotopes can be considered as constant for the 10 years. In contrast, based on the half-lives of 2.0648 y for ^134^Cs and 30.17 y for ^137^Cs, 96.5 wt% of ^134^Cs and 20.6 wt% of ^137^Cs had decayed 10 years after the accident. In terms of the proportion to the total weight of Cs, it can be concluded that 11.6 wt% of Cs decayed to Ba. In addition, the β and γ rays emitted during this decay may have damaged the glass structure of the particles. CsMPs that remained in the environment for several years after the accident may have become more fragile owing to physical damage, such as weathering^39^. Alternatively, chemical reactions between the metal elements in the CsMPs and oxygen in the air might have occurred. Yamaguchi et al.^9^ and Kogure et al.^27^ reported that the distribution of Cs in spherical CsMPs was only two to three times different between the inside and outside of the particles, although there were individual differences among CsMPs. Therefore, the effect of Cs decay for localized structural collapse was presumed to be negligible if the Cs was uniformly decayed. In contrast, non-spherical CsMPs derived from FDNPP Unit 2 exhibited a heterogeneous elemental distribution, as observed on the surface using SEM^3^. Some surface areas of non-spherical CsMPs showed aggregation of Cs over 10-times those of other spherical CsMPs, whereas other areas had no Cs^3^. The localized conversion of 11.6 wt% Cs to Ba in areas with Cs aggregates might lead to localized structural collapse within the CsMP, potentially resulting in the localized destruction of the overall structure of the CsMP. It is also suggested that the radiocesium in CsMPs such as Ushiku-20110320-B discovered in this study might have unevenly decayed. Therefore, long-term experiments are required to investigate the physical fragility of CsMPs as well as their characteristics and environmental impact, for example, by creating simulated glass particles.

## Conclusions

After the FDNPP accident, CsMPs that arrived in the Kanto region by dry deposition on the ground were collected using the wiping method and analyzed using gamma-ray measurements and SEM–EDS to reveal the characteristics of the CsMPs that had spread in the Kanto region. In the chaos following the huge earthquake and the accident, the method applied was appropriate. Using this method, 106 CsMPs were obtained. The two CsMPs found in Ushiku City were concluded to be two spherical Type A CsMPs stuck together. The remaining 104 were typical spherical CsMPs. Although typical spherical CsMPs did not show any change in luminescent spots after ultrasonic cleaning with acetone nor DMF, these CsMPs underwent mechanical particle breakage. These CsMPs may undergo mechanical breakage in the order of approximately 10 years (and there is concern about the impact of this on environmental radiation). This study provides novel insight into the possibility that CsMPs were depsited in the Kanto region and have been physically fragile for approximately 10 years.

### Supplementary Information

Supplementary Information.

## Data Availability

The datasets generated and/or analyzed during the current study are available from the corresponding author on reasonable request.
