# Supplementary material for: Radiocesium-bearing microparticles found in dry deposition fallout samples immediately after the Fukushima nuclear accident in the Kanto region, Japan
Source: Sci Rep. 2023 Dec 9;13:21826. doi: 10.1038/s41598-023-49158-2 (PMC10710400; doi:10.1038/s41598-023-49158-2)
Supplement: Supplementary file 1 — Supplementary Information. [file 41598_2023_49158_MOESM1_ESM.pdf]

Supplementary information

Radiocesium-bearing microparticles found in dry deposition fallout samples immediately after the Fukushima nuclear accident in the Kanto region, Japan

Authors

Yuki Takaku[1,2], Shogo Higaki[2]*, Masahiro Hirota[3] and Hiroyuki Kagi[1]

Affiliations

[1]Geochemical Research Center, Graduate School of Science, The University of Tokyo, JAPAN.

[2]Isotope Science Center, The University of Tokyo, JAPAN.

[3]Research Center for Supports to Advanced Sciences, Shinshu University, JAPAN.

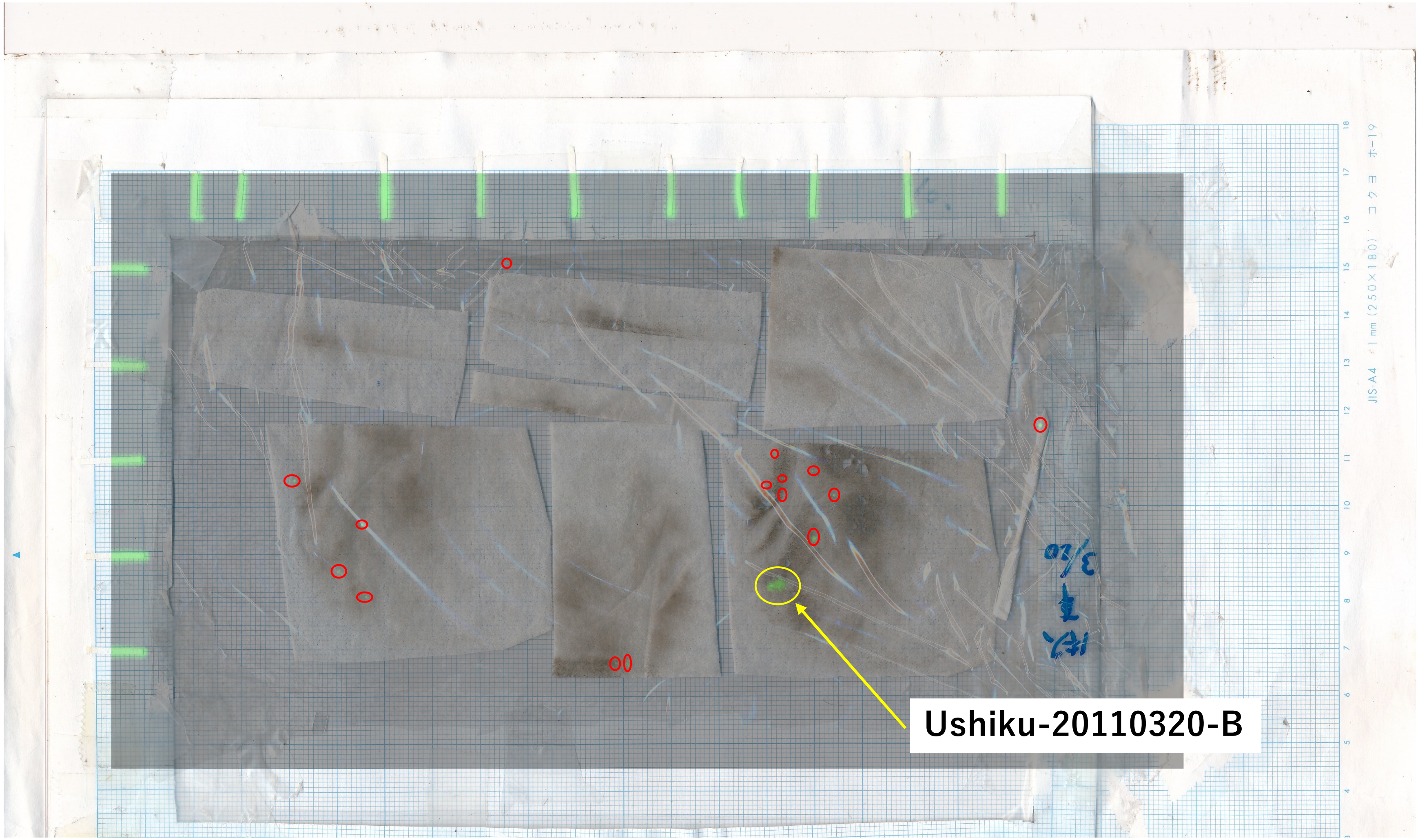

Supplementary Fig. S1 Large asymmetric bright spots discovered on the tissue paper of Ushiku-20110320. The CsMP was named Ushiku-20110320-B according to the naming rules.

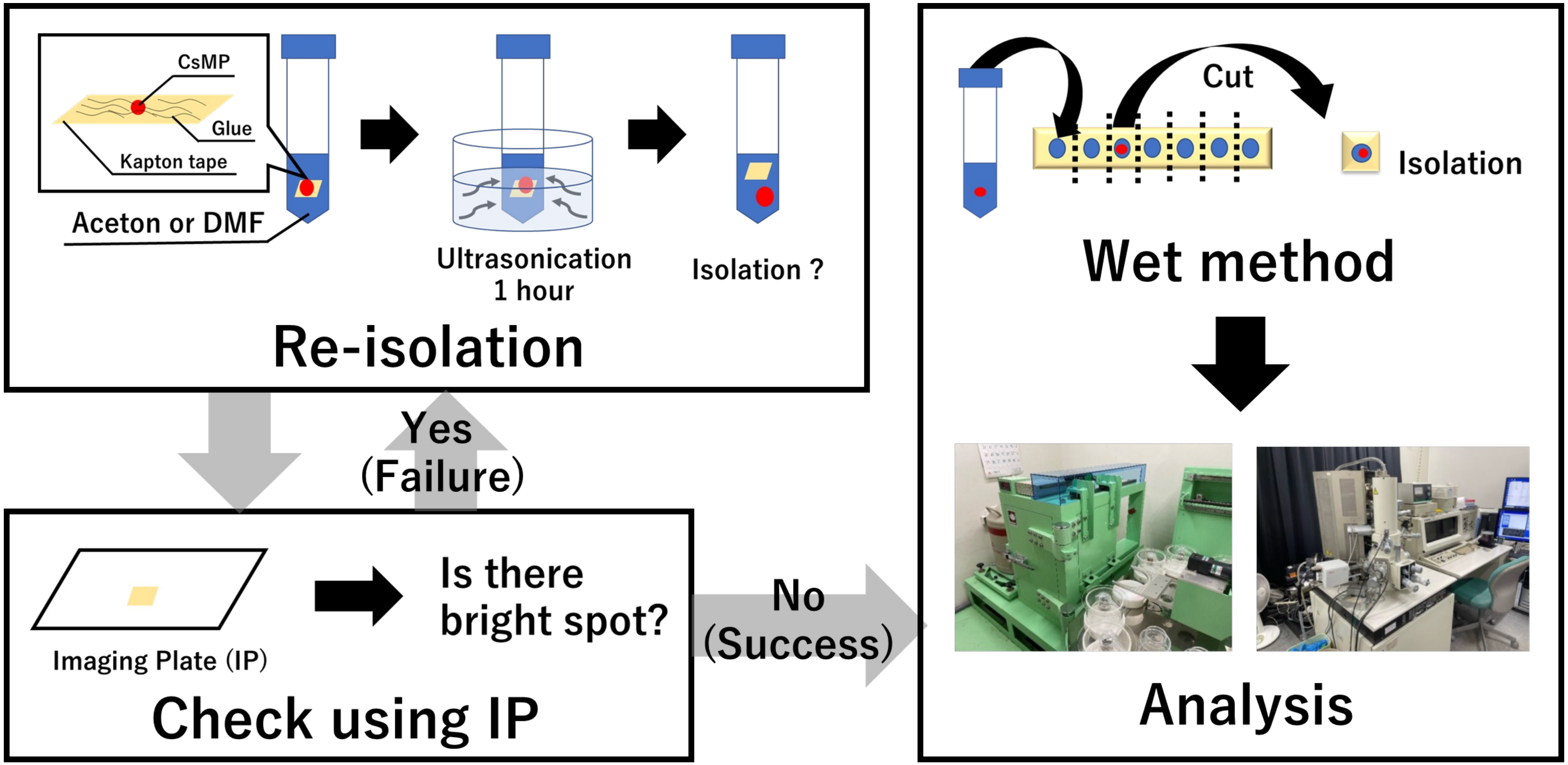

Supplementary Fig. S2 Flow of the rewetting method using acetone or DMF, which was attempted for the re-isolation of CsMP

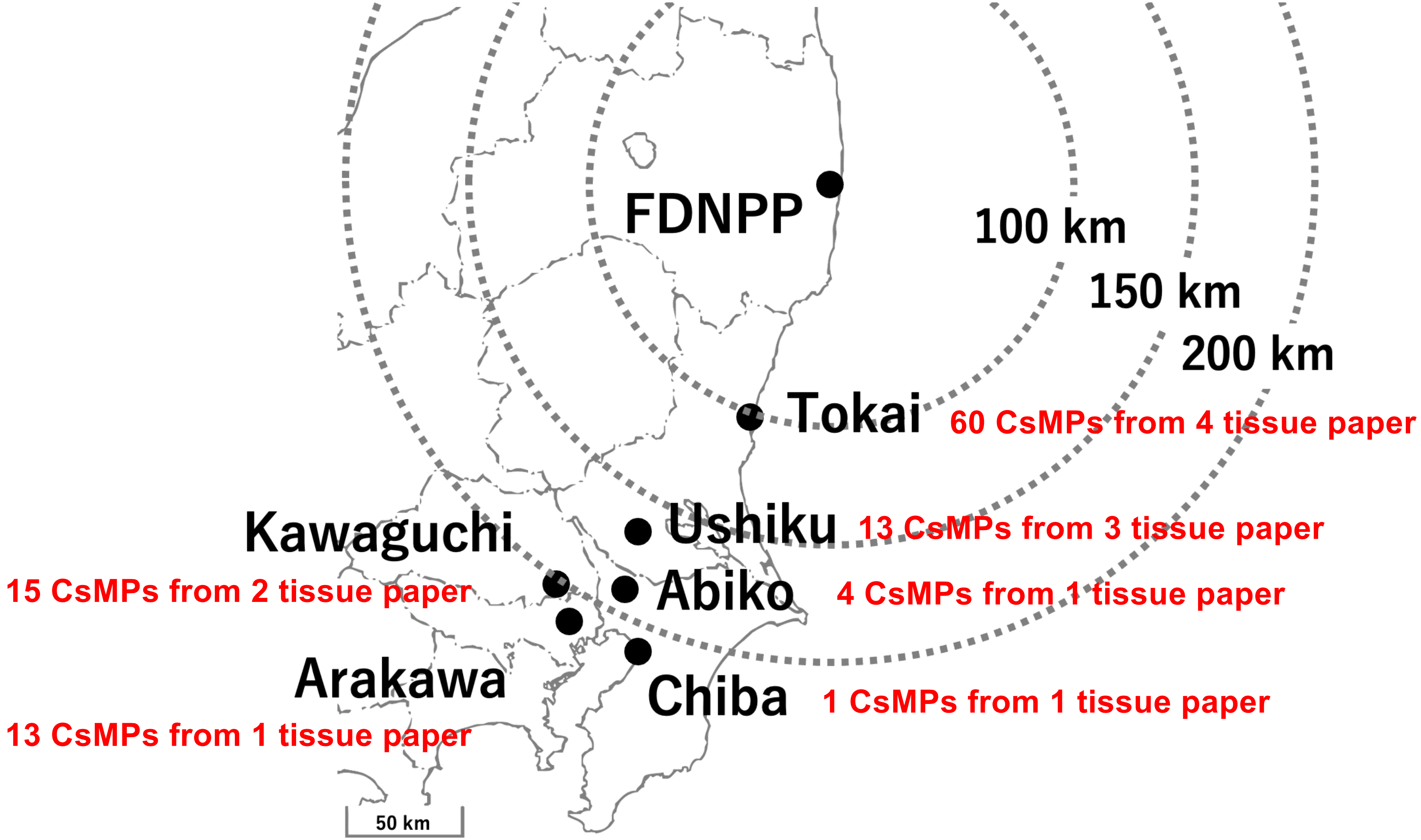

Supplementary Fig. S3 Sampling locations and found number of CsMP to total sample in this study, created using Adobe Illustrator software (version 28.0) and Microsoft PowerPoint (version 16.79). Circles indicate distances from the FDNPP.

Supplementary Table S1 Radioactivity of CsMPs found in this study. The measurement error shows 1σ.

Sample	At the accident			At the measurement	
	^{134}Cs [Bq]	^{137}Cs [Bq]	^{134}Cs/^{137}Cs	^{134}Cs [Bq]	^{137}Cs [Bq]
Tokai1-20110320-A	0.293±0.122	0.337±0.004	0.870±0.362	0.00874±0.00363	0.265±0.003
Tokai1-20110320-B	—	0.133±0.005	—	not detected	0.105±0.004
Tokai1-20110320-C	—	0.197±0.006	—	not detected	0.154±0.004
Tokai1-20110320-D	0.456±0.148	0.503±0.005	0.91±0.29	0.0133±0.0043	0.395±0.004
Tokai1-20110320-E	—	0.229±0.005	—	not detected	0.180±0.004
Tokai1-20110320-F	—	0.0796±0.0036	—	not detected	0.0629±0.0028
Tokai1-20110320-G	0.734±0.196	0.691±0.007	1.06±0.28	0.0236±0.0062	0.546±0.006
Tokai1-20110320-H	—	0.313±0.005	—	not detected	0.247±0.004
Tokai1-20110320-I	—	0.206±0.005	—	not detected	0.162±0.004
Tokai1-20110320-J	—	0.195±0.004	—	not detected	0.148±0.003
Tokai1-20110320-K	—	0.121±0.004	—	not detected	0.0948±0.0032
Tokai1-20110320-L	—	0.0737±0.0036	—	not detected	0.058±0.003
Tokai1-20110320-M	—	0.260±0.004	—	not detected	0.204±0.003
Tokai1-20110320-N	0.829±0.196	0.682±0.007	1.22±0.29	0.0263±0.0062	0.538±0.006
Tokai1-20110320-O	1.24±0.25	1.09±0.01	1.14±0.23	0.0386±0.0076	0.858±0.007
Tokai1-20110320-P	—	0.0734±0.0025	—	not detected	0.0577±0.0019
Tokai1-20110320-Q	0.321±0.114	0.281±0.004	1.14±0.41	0.00945±0.00336	0.202±0.003
Tokai1-20110320-R	0.325±0.104	0.192±0.003	1.69±0.54	0.00907±0.00290	0.150±0.003
Tokai1-20110320-S	—	0.145±0.004	—	not detected	0.114±0.003
Tokai1-20110320-T	—	0.124±0.004	—	not detected	0.0973±0.0032
Tokai1-20110320-U	0.929±0.172	0.855±0.007	1.09±0.20	0.0297±0.0055	0.675±0.005
Tokai2-20110320-A	0.607±0.182	0.655±0.006	0.927±0.277	0.0163±0.0049	0.511±0.005
Tokai2-20110320-B	1.01±0.31	0.717±0.007	1.41±0.43	0.0173±0.0053	0.542±0.005
Tokai2-20110320-C	1.50±0.26	1.30±0.01	1.15±0.19	0.0398±0.0067	1.01±0.01
Tokai2-20110320-D	—	0.109±0.0033	—	not detected	0.0848±0.0022
Tokai2-20110320-E	2.62±0.33	2.41±0.01	1.09±0.14	0.0712±0.0090	1.88±0.01
Tokai2-20110320-F	0.421±0.163	0.460±0.005	0.915±0.355	0.0107±0.0041	0.357±0.004
Tokai2-20110320-G	0.562±0.169	0.538±0.005	1.04±0.31	0.0147±0.0044	0.419±0.004
Tokai2-20110320-H	—	0.147±0.004	—	not detected	0.112±0.003
Tokai2-20110320-I	—	0.147±0.006	—	not detected	0.111±0.004
Tokai2-20110320-J	—	0.208±0.005	—	not detected	0.162±0.004
Tokai2-20110320-K	—	0.154±0.006	—	not detected	0.121±0.004
Tokai2-20110320-L	—	0.658±0.007	—	not detected	0.511±0.006
Tokai2-20110320-M	—	0.106±0.004	—	not detected	0.0827±0.0031
Tokai2-20110320-N	0.531±0.160	0.271±0.005	1.95±0.59	0.0144±0.0043	0.212±0.004
Tokai2-20110320-O	—	0.275±0.005	—	not detected	0.216±0.004
Tokai2-20110320-P	—	0.0451±0.038	—	not detected	0.0352±0.0030
Tokai2-20110320-Q	—	0.230±0.005	—	not detected	0.180±0.004
Tokai2-20110320-R	—	0.264±0.005	—	not detected	0.207±0.004
Tokai2-20110320-S	—	0.152±0.005	—	not detected	0.115±0.004
Tokai2-20110320-T	—	0.205±0.004	—	not detected	0.155±0.003
Tokai2-20110320-U	0.465±0.142	0.399±0.005	1.17±0.36	0.0128±0.0039	0.311±0.004
Tokai3-20110320-A	0.462±0.103	0.316±0.005	1.46±0.33	0.0206±0.0046	0.256±0.004
Tokai3-20110320-B	0.414±0.173	0.385±0.006	1.08±0.45	0.0119±0.0050	0.302±0.005
Tokai3-20110320-C	2.06±0.22	1.78±0.01	1.16±0.12	0.0923±0.0097	1.43±0.01
Tokai3-20110320-D	0.648±0.232	0.573±0.007	1.13±0.41	0.0162±0.0058	0.445±0.005
Tokai3-20110320-E	—	0.163±0.003	—	not detected	0.126±0.003
Tokai3-20110320-F	0.798±0.190	0.814±0.006	0.980±0.234	0.0226±0.0054	0.637±0.005
Tokai3-20110320-G	—	0.227±0.006	—	not detected	0.176±0.004
Tokai3-20110320-H	0.724±0.259	0.551±0.008	1.31±0.47	0.0178±0.0064	0.427±0.006
Tokai3-20110320-I	0.522±0.100	0.488±0.005	1.07±0.20	0.0230±0.0040	0.393±0.004
Tokai3-20110320-J	3.41±0.31	3.14±0.02	1.09±0.10	0.151±0.014	2.53±0.01
Tokai3-20110320-K	0.729±0.205	0.602±0.007	1.21±0.34	0.0210±0.0059	0.471±0.006
Tokai3-20110320-L	0.250±0.136	0.275±0.004	0.909±0.495	0.00611±0.00332	0.213±0.003
Tokai3-20110320-M	—	0.399±0.006	—	not detected	0.310±0.005
Tokai3-20110320-N	1.25±0.33	1.06±0.01	1.18±0.31	0.0305±0.0080	0.821±0.008
Tokai4-20110320-A	0.674±0.164	0.699±0.006	0.965±0.235	0.0206±0.0050	0.550±0.005
Tokai4-20110320-B	0.436±0.166	0.486±0.005	0.90±0.34	0.0111±0.0042	0.378±0.004
Tokai4-20110320-C	1.48±0.27	1.25±0.01	1.19±0.21	0.0455±0.0081	0.980±0.008

Tokai4-20110320-D	—	0.0793±0.0026	—	not detected	0.0620±0.0020
Ushiku-20110317-A	—	0.110±0.004	—	not detected	0.0881±0.0031
Ushiku-20110317-B	0.451±0.124	0.389±0.006	1.16±0.32	0.0180±0.0049	0.312±0.005
Ushiku-20110318-A	1.17±0.19	1.05±0,01	1.11±0.19	0.0458±0.0076	0.842±0.007
Ushiku-20110318-B	0.556±0.131	0.428±0.006	1.30±0.31	0.0219±0.0052	0.342±0.005
Ushiku-20110318-C	0.473±0.132	0.460±0.006	1.03±0.29	0.0190±0.0053	0.369±0.005
Ushiku-20110318-D	0.236±0.101	0.232±0.004	1.02±0.43	0.00737±0.00314	0.183±0.003
Ushiku-20210320-A	2.15±0.23	2.01±0.01	1.07±0.11	0.0778±0.0083	1.60±0.01
Ushiku-20210320-B	6.59±0.54	5.81±0.03	1.13±0.09	0.0323±0.0027	4.72±0.03
Ushiku-20210320-C	0.904±0.128	0.788±0.007	1.15±0.16	0.0417±0.0059	0.638±0.005
Ushiku-20210320-D	—	0.245±0.006	—	not detected	0.186±0.005
Ushiku-20210320-E	—	0.499±0.008	—	not detected	0.378±0.006
Ushiku-20210320-F	—	0.197±0.005	—	not detected	0.155±0.004
Ushiku-20210320-G	0.314±0.097	0.311±0.004	1.01±0.31	0.0115±0.0036	0.248±0.003
Abiko-20110321-A	0.301±0.115	0.247±0.005	1.22±0.47	0.0109±0.0042	0.197±0.004
Abiko-20110321-B	0.694±0.169	0.629±0.007	1.10±0.27	0.0248±0.0060	0.500±0.006
Abiko-20110321-C	1.88±0.37	1.74±0.01	1.08±0.19	0.0673±0.0117	1.39±0.01
Abiko-20110321-D	—	0.329±0.007	—	not detected	0.249±0.005
Chiba-20110321	0.278±0.114	0.274±0.005	1.02±0.42	0.0106±0.0044	0.219±0.004
Kawaguchi-20110316-A	0.403±0.145	0.407±0.006	0.990±0.356	0.0140±0.005	0.323±0.005
Kawaguchi-20110316-B	0.473±0.142	0.411±0.006	1.15±0.35	0.0168±0.0050	0.327±0.005
Kawaguchi-20110316-C	0.373±0.134	0.427±0.006	0.873±0.313	0.0143±0.0050	0.341±0.005
Kawaguchi-20110316-D	—	0.207±0.05	—	not detected	0.164±0.004
Kawaguchi-20110316-E	—	0.106±0.003	—	not detected	0.0840±0.0022
Kawaguchi-20110316-F	0.177±0.134	0.163±0.005	1.09±0.82	0.00605±0.00457	0.129±0.004
Kawaguchi-20110316-G	0.721±0.183	0.649±0.007	1.11±0.28	0.0240±0.0061	0.514±0.006
Kawaguchi-20110316-H	0.536±0.160	0.467±0.006	1.15±0.34	0.0177±0.0053	0.369±0.005
Kawaguchi1-20110320-A	—	0.226±0.005	—	not detected	0.179±0.00$_4$
Kawaguchi1-20110320-B	0.354±0.125	0.442±0.005	0.800±0.284	0.0116±0.0041	0.350±0.004
Kawaguchi1-20110320-C	—	0.181±0.004	—	not detected	0.143±0.00$_4$
Kawaguchi1-20110320-D	0.546±0.330	0.539±0.007	1.01±0.61	0.00960±0.00581	0.409±0.005
Kawaguchi2-20110320-A	—	0.132±0.003	—	not detected	0.104±0.002
Kawaguchi2-20110320-B	—	0.0717±0.0025	—	not detected	0.0565±0.0020
Kawaguchi2-20110320-C	—	0.123±0.003	—	not detected	0.0966±0.0023
Arakawa-20110317-A	0.872±0.140	0.828±0.006	1.05±0.17	0.0340±0.0055	0.663±0.005
Arakawa-20110317-B	0.803±0.143	0.800±0.006	1.00±0.18	0.0301±0.0054	0.638±0.005
Arakawa-20110317-C	0.308±0.077	0.199±0.003	1.55±0.39	0.0119±0.0030	0.159±0.003
Arakawa-20110317-D	0.394±0.096	0.358±0.004	1.10±0.27	0.0155±0.0038	0.286±0.003
Arakawa-20110317-E	0.184±0.081	0.249±0.004	0.74±0.33	0.0073±0.0032	0.199±0.003
Arakawa-20110317-F	0.397±0.121	0.455±0.005	0.874±0.266	0.0136±0.0041	0.360±0.004
Arakawa-20110317-G	0.559±0.121	0.567±0.005	0.986±0.214	0.0212±0.0046	0.453±0.004
Arakawa-20110317-H	0.290±0.079	0.191±0.003	1.52±0.41	0.0107±0.0029	0.152±0.003
Arakawa-20110317-I	0.126±0.080	0.204±0.003	0.619±0.393	0.00471±0.00299	0.163±0.003
Arakawa-20110317-J	0.779±0.127	0.621±0.006	1.26±0.21	0.0294±0.0048	0.496±0.004
Arakawa-20110317-K	—	0.116±0.003	—	—	0.0924±0.0023
Arakawa-20110317-L	0.916±0.165	0.765±0.008	1.20±0.22	0.0364±0.0066	0.613±0.006
Arakawa-20110317-M	0.324±0.100	0.291±0.004	1.11±0.34	0.0112±0.0034	0.231±0.003
